# A statistical investigation of normal regional intra-subject heterogeneity of brain metabolism and perfusion by F-18 FDG and O-15 H_2_O PET imaging

**DOI:** 10.1186/1471-2385-6-4

**Published:** 2006-07-12

**Authors:** Ching-yee Oliver Wong, Joseph Thie, Marianne Gaskill, Richard Ponto, Jack Hill, Hai-yan Tian, Helena Balon, Dafang Wu, Darlene Fink-Bennett, Conrad Nagle

**Affiliations:** 1 Nuclear Medicine, William Beaumont Hospital, Royal Oak, MI, USA; 2 Graduate School of Medicine, University of Tennessee Medical Center, Knoxville, TN, USA; 3 Medical Information Services, William Beaumont Hospital, Royal Oak, MI, USA

## Abstract

**Background:**

The definite evaluation of the regional cerebral heterogeneity using perfusion and metabolism by a single modality of PET imaging has not been well addressed. Thus a statistical analysis of voxel variables from identical brain regions on metabolic and perfusion PET images was carried out to determine characteristics of the regional heterogeneity of F-18 FDG and O-15 H_2_O cerebral uptake in normal subjects.

**Methods:**

Fourteen normal subjects with normal CT and/or MRI and physical examination including MMSE were scanned by both F-18 FDG and O-15 H_2_O PET within same day with head-holder and facemask. The images were co-registered and each individual voxel counts (Q) were normalized by the gloabl maximal voxel counts (M) as R = Q/M. The voxel counts were also converted to z-score map by z = (Q - mean)/SD. Twelve pairs of ROIs (24 total) were systematically placed on the z-score map at cortical locations 15-degree apart and identically for metabolism and perfusion. Inter- and intra-subject correlation coefficients (r) were computed, both globally and hemispherically, from metabolism and perfusion: between regions for the same tracer and between tracers for the same region. Moments of means and histograms were computed globally along with asymmetric indices as their hemispherical differences.

**Results:**

Statistical investigations verified with data showed that, for a given scan, correlation analyses are expectedly alike regardless of variables (Q, R, z) used. The varieties of correlation (r's) of normal subjects, showing symmetry, were mostly around 0.8 and with coefficient of variations near 10%. Analyses of histograms showed non-Gaussian behavior (skew = -0.3 and kurtosis = 0.4) of metabolism on average, in contrast to near Gaussian perfusion.

**Conclusion:**

The co-registered cerebral metabolism and perfusion z maps demonstrated regional heterogeneity but with attractively low coefficient of variations in the correlation markers.

## Background

Cerebral perfusion SPECT and metabolic PET imaging has been widely used for evaluation of functional abnormalities in patients with dementia and epilepsy [1,2]. CT or MRI is usually employed in the evaluation of brain structural abnormalities and may clearly delineate the anatomic extent of brain diseases. The general internists and neurologists are often confronted with diagnostic difficulties when CT or MRI is normal. Then functional imaging using perfusion SPECT or metabolic PET is commonly used. Flow and metabolism are expected to be coupled in normal state of brain [3] and SPECT and PET have been used interchangeably in most clinical situations depending on the availability of the equipment. However, previous quantitative studies using PET and SPECT have demonstrated the regional heterogeneity of perfusion and metabolism in dementia patients [4,5]. Although the SPECT and PET images had been reconstructed in similar way in the prior studies, the definite evaluation of this regional heterogeneity using perfusion and metabolism by a single modality of PET imaging has not been addressed in detail. To better define the relationship among voxel variables, a statistical analysis of voxels from identical brain regions on metabolic and perfusion PET images was carried out. The purpose of this study is to determine the statistical characteristics of regional heterogeneity of Fluorine-18-2-fluoro-2-deoxy-D-glucose (F-18 FDG) and Oxygen-15-water (O-15 H_2_O) cerebral uptakes in normal subjects.

## Methods

**Figure 1:**
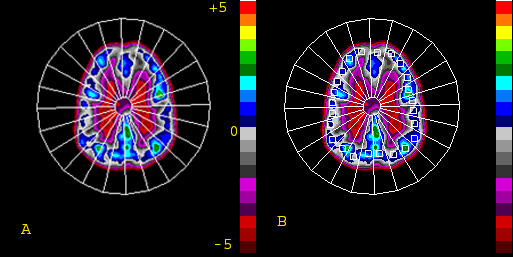
The computer generated 15-degree sectors (A) and ROIs (B) on the PET images displayed in 20 color steps with the mean (z = 0) at the center and each interval representing 0.5 standard deviation ranging from z = -5.0 to +5.0.

Fourteen subjects (average age = 42 ± 13, range = 19 – 56 years, 7 males and 7 females) with normal CT/MRI and neurological examination who had both F-18 FDG and O-15 water PET imaging were used to generate normal brain profile for statistical analysis. All patients had CT and/or MRI. As the PET studies were considered part of the clinical evaluation, the ethical approval for the study was granted by the institutional review board. The injections of tracers and acquisition of PET images were performed in a quiet environment with the eyes and ears open. The head was secured in standard position by a holder with a facemask. Cerebral perfusion PET images were first acquired by a Siemens 951 PET camera for a total of 1 min in 2-D mode thirty-five seconds after injection of an average of 3170 ± 195 MBq O-15 water to avoid excessive blood pool activity and assembled into frames having a 256 by 256 matrix with a pixel size of 0.117 cm. After the perfusion PET scan, cerebral metabolic PET scan was acquired by using the same camera without movement in between the two scans in the same matrix on the same day for a total of 20 min also in 2-D mode one hour after injection of an average of 369 ± 27 MBq F-18 FDG. After acquisition, the images were processed by linear back projection with a Hanning filter at cut-off frequencies of 0.4 and 0.5 cycles per pixel for O-15 water and F-18 FDG respectively and a uniform attenuation coefficient 0.095 cm^-1^. Even the field of view was only 10.5 cm, most of the patients were fitted into one bed position. The perfusion and metabolic PET images were then submitted for co-registration between two sets of PET images using AIR program by Woods RP, et al [6] for further image alignment for possible minor change in position during acquisition between perfusion and metabolic images. The images were displayed conventionally as a ratio (R) of each individual voxel counts (Q) to the maximal voxel counts (M) of the entire brain by: R = Q/M. The co-registered images (voxel = 0.827 cm^3^) were finally transformed into z-score map displaying as standard deviations from each individual mean global perfusion or metabolism of the entire brain by using the method by Wong CYO, et al [7]: z = (Q - mean)/SD. Averaged z-scores from multiple cortical regions of interest (ROIs) with a size of 6.619 cm^3 ^placed identically on the cortices of the co-registered transverse perfusion and metabolic PET images within the center of 15-degree sectors (Fig. 1A) which were determined automatically by computer centered at the midline across the anterior to posterior brain and the vertical line bisecting right and left brain of the transverse slice of perfusion and metabolism PET images orientated along the temporal long axis. Two identical coregistered transverse slices from perfusion and metabolic PET images across the the superior aspect of the basal ganglia and the primary visual cortex were used to obtain from each slice the twelve pairs of ROIs (24 total) distributed over the frontal, parietal and temporo-occipital cortices (Fig. 1B). In order to cover other areas of the brain, the regions of interest same size as in the above profile analysis were placed symmetrically on the caudate and thalamic, mesial anterior, lateral, and posterior temporal, inferior frontal, pons, vermis and cerebellar regions. The averaged z-scores of the corresponding right and left side perfusion and metabolism were compared by paired t-tests with vigorous Bonferroni correction for the 23 comparisons (Table 1).

**Table 1:** The averaged z-scores of right and left embolism and perfusion across major brain regions.

		***Metabolism (z)***		***Perfusion (z)***		***Paired-t (Bonferroni)***	
**ROI#**	**Regions**	**Mean**	**S.D.**	**Mean**	**S.D.**	**p-value**	**<0.0022**

1	Cingular	0.8846	0.6735	0.4986	0.70080	0.149366	no
2	Superior Frontal	0.7318	0.5423	0.0142	0.34289	0.000385	yes
3	Superior Frontal	0.8912	0.3859	0.0289	0.31127	0.000001	yes
4	Superior Frontal	1.0526	0.4551	0.4382	0.36553	0.000586	yes
5	Superior Frontal	0.8950	0.5497	0.3445	0.37645	0.005150	no
6	Superior Frontal	0.7323	0.5901	0.5638	0.64511	0.477221	no
7	Sensorimotor	1.6202	0.5458	1.1141	0.47482	0.014671	no
8	Paritooccipital	0.8830	0.4685	0.7357	0.54474	0.449990	no
9	Paritooccipital	0.3831	0.6245	0.4224	0.55062	0.861338	no
10	Paritooccipital	-0.2213	0.7214	-0.4628	0.58784	0.340849	no
11	Occipitotemperal	-1.0677	0.8393	-1.2533	0.51900	0.489200	no
12	Visual	2.2786	0.3254	1.8334	0.50429	0.010973	no
13	caudate	-0.2671	0.5250	-0.6453	0.86906	0.177700	no
14	thalamic	0.4491	0.7469	1.2163	0.66755	0.008191	no
15	mesial temporal	-1.2518	0.8707	0.5084	0.60956	0.000002	yes
16	anterior temporal	-0.8298	0.7871	0.2296	0.68733	0.000819	yes
17	lateral temporal	-0.5011	0.6355	0.2198	0.63933	0.005997	no
18	posterior temporal	-0.2170	0.5528	-0.1331	0.43038	0.658018	no
19	inferior frontal	0.2181	0.5461	0.4214	0.62663	0.368604	no
20	inferior frontal	-0.2259	0.5707	-0.6542	0.48945	0.042897	no
21	pons	-1.4030	0.7198	-0.0206	0.88517	0.000125	yes
22	vermis	-0.1678	0.7890	1.1355	0.68684	0.000086	yes
23	cerebellar	-0.0092	0.4359	1.1268	0.83006	0.000210	yes

One approach taken to describe the statistical behavior of the combination of metabolism and perfusion scans was done by computing varieties of Pearson correlation coefficients (r) in which items paired were varied as well as the product averaging computation being performed either over regions or over patients. Intra-subject correlation coefficients (r_j_) of each individual normal subject (j) were computed among regions between hemispheres (h) of the metabolism (m) or perfusion (p) PET images as r_jmh_or r_jph _and between metabolish-perfusion (m-p) pairings of regions of these two sets of co-registered PET images over the entire brain and right (r) and left (l) hemispheres for each individual subject (j) as respectively r_jmp_, r_jmpr _and r_jmpl_.

Inter-subject correlation coefficients (r_i_) of each individual region (i) were similarly computed between each individual region (i) and the global means of all regions of a paricular normal subjects' metabolism (m) or perfusion (p) as respectively r_im _or r_ip_. Here the first variable is from each individual region from a patient (14 data points in total) and the second variable is the global mean of all the 24 regions of a particular individual (thus another 14 data points for the 14 subjects). The inter-subject correlation between m-p pairings of each particular region across the normal subjects is also obtained as r_imp_. The mean metabolism and perfusion of all subjects for each particular region was used to generate regional profile.

Another statistical approach also taken to characterize the images was by quantifying the spatial heterogeneity of the histogram of activities. Here the popular quantifiers skew and kurtosis describing respectively the asymmetry and the extent of symmetrical departure from a Gaussian shape were employed. The statistically computed standard errors of skew and kurtosis together with the separation of the 2 values were used for testing its significance.

The coefficients of variations of the parameters obtained within the normal subjects were also computed. A p-value of less than 0.05 was considered significant in all tests.

## Results

Statistical investigations verified with data showed that, for a given scan, correlation analyses are as expected alike regardless of variables (Q, R, z) used. Thus, z values were used for the various statistical analyses. The images were displayed in 20 color steps (7) with the mean (z = 0) at the center and each interval representing 0.5 standard deviation ranging from z = -5.0 to +5.0 (Fig. 1).

### 1. Analysis of correlation within the same tracer

The averages of the intra-subject coefficients of correlation (r_j_'s) of the individual normal subjects' right and left hemispheric metabolism and perfusion (r_jmh _and r_jph_) were 0.80 ± 0.10 and 0.76 ± 0.09 respectively, which were not significantly different. The averages of the inter-subject coefficients of correlation (r_i_'s) of a particular region of across normal subjects' metabolism and perfusion against their respective global means of all regions from each individual subject (r_im _and r_ip_) were 0.80 ± 0.09 and 0.80 ± 0.05 respectively, which were again not significantly different.

### 2. Analsysis of correlation between tracers

**Figure 2:**
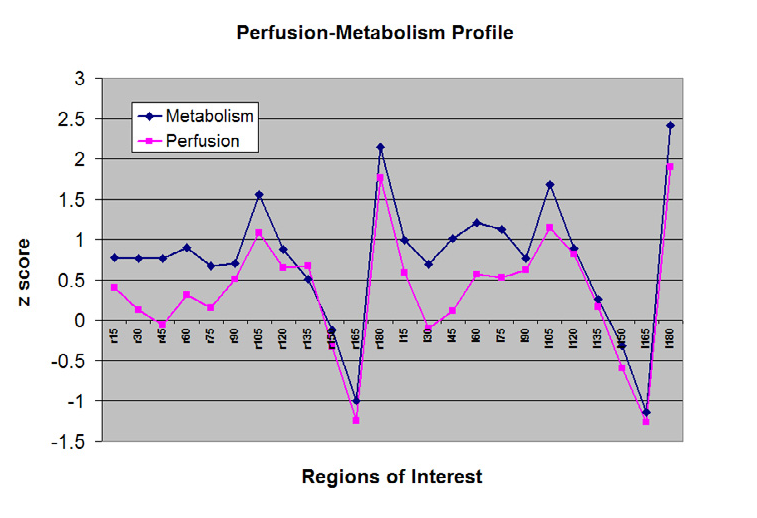
The perfusion and metabolism profile from regions of interest in Figure 1. Legends: r = right, l = left, numbers indicated the degree of the sector with 0 at the anterior (frontal area) and 180 at the posterior (occipital area) parts of the brain.

The average intra-subject coefficients of correlation between metabolism and perfusion, r_jmp_'s for individual subjects of the entire, right (r) side and left (l) side of the brain were r_jmp _= 0.77 ± 0.07, r_jmpr _= 0.76 ± 0.08, and r_jmpl _= 0.77 ± 0.08 respectively with no statistically differences between right and left sides. However, the average inter-subject coefficients of correlation (r_imp_) for a particular region between perfusion and metabolism across the subjects was only 0.42 ± 0.22, which was significantly different than that of the intra-subject correlation of m-p pairings (r_jmp_) for individual normal subjects (p < 0.0005). The discrepancy was mainly in the frontal area (Fig. 2)

**Figure 3:**
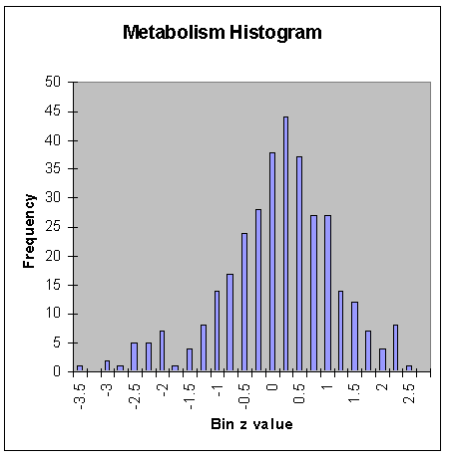
Histogram of z values of metabolism from all 24 regions in all 14 normal subjects. Note the substantial negative (leftward) skew = -0.28 and the shape being leptokurtic with kurtosis = 0.39.

**Figure 4:**
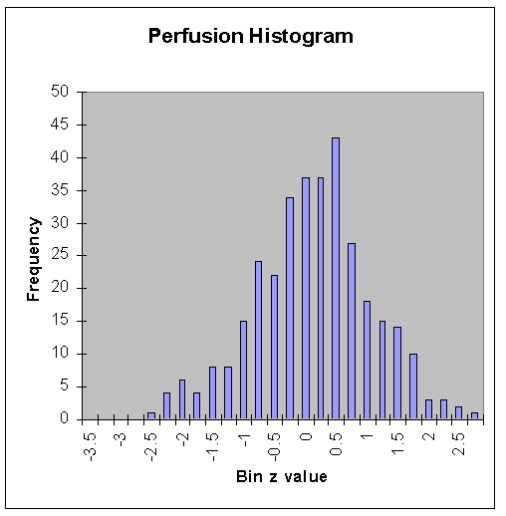
Histogram of z values of perfusion from all 24 regions in all 14 normal subjects. Note the minimal negative (leftward) skew = -0.07 and the shape also being minimally platykurtic or near-Gaussian with kurtosis = -0.06.

### 3. Analysis of voxel histograms

Analyses of metabolism and perfusion histograms showed the non-Gaussian behavior of metabolism on average, with skew = -0.28 and kurtosis = 0.39 (Fig. 3), in contrast to near Gaussian (zero values for skew and kurtosis) perfusion with skew = -0.07 and kurtosis = -0.06 (Fig. 4).

### 4. Analysis of regional perfusion and metabolism profiles

Besides the regional differences differences in the frontal areas with higher metabolism than perfusion, the paired t-tests with Boferroni corrections suggested that other major regional differences were in the mesial temporal and cerebellare regions with the opposite results of the frontal areas, viz. higher perfusion than metabolism in the latter two regions (Table 1).

## Discussion

In order to evaluate fully the normal regional heterogeneity of cerebral imaging, the current study employed both perfusion and metabolic PET imaging, instead of the less preferable SPECT perfusion and PET metabolism imaging [4,5]. A statistical analysis of normal subjects with functional imaging by a pure metabolism and pure perfusion tracer using PET confirmed the previous observations using PET and SPECT [4,5]. The present study was designed to derive a clinically useful profile of regional heterogeneity by statistical investigation of perfusion and metabolic PET images in normal subjects. No attempt was made to quantify such differences in perfusion and metabolic parameters by absolute terms or to try to explain the complex relationship between cerebral flow and volume of distribution [3,8]. As the images were acquired and processed in the same way as the routine clinical studies, the heterogeneity between cortical uptake in perfusion and metabolic PET images in normal subjects may explain partially the differences in sensitivity for clinical diagnosis on dementia or epilepsy patients with separate modalities of perfusion SPECT and metabolic PET images. The study echoed the previous observations [4,5] that loss of heterogeneity between metabolism and perfusion would potentially be a marker of disease or specially the severity of dementia. The coefficients of variations were smaller than that from prior studies from PET and SPECT analysis [4,5], which might result from the use single modality PET for both perfusion and metabolism in the current study.

The study was obviously limited in its inter-subject comparsions as the ROIs were all determined by computer generation of sectors. Besides the differences in the hematocrit, there may not be exact anatomical correlation for each individual sector of the brain among different subjects. Only a more tedious method of co-registration with MRI into Talairach coordinate space will ensure the exact comparison. However, the study was mainly designed and focused on the intra-subject comparisons between perfusion and metabolism PET images and addressing individual subjects' regional heterogeneity between these two PET scans. Thus, for additional inter-subject comparions, the global mean perfusion and metabolism of each region of all subjects from the 15-degree sector ROIs (Fig. 1) and other ROIs placed elsewhere in the rest of the brain (Fig. 2 and Table 1) was used to generate a regional profile. Despite all the limitations of individual subjects' variation, the major discrepancy of lower perfusion than metabolism was found to be in the frontal region (Fig. 2), similar to previous observation [9]. The profiles were quite similar in the occipital region, which was different than prior observation [10], with limitations due to the potential age differences of the study population [9]. The opposite observations of higher perfusion than metabolism were noted over temporal and cerebellar regions (Table 1), similar to the previous studies using complex absolute quantification by dynamic PET studies [11].

Image display systems are commonly default to intensity settings determined by the most intense portion of the image. In PET brain imaging with uniform attenuation coefficient, this is typically found in the visual cortex. Due to different degree of visual activity, clinical quantification usually employs the relatively stable cerebellum or entire brain counts as the reference. The present study used z-transformation to avoid potential errors in the commonly used semi-quantitative of perfusion or metabolism scans using cerebellum or maximal voxel count region as the reference. The individualized z-normalization was used to exploit the cerebral heterogeneity between metabolism and perfusion within an individual subject. However, it was shown in the study that there was statistical equivalence in the analysis of cerebral heterogeneity no matter which normalization methods were used (ratio or z-score map). Thus, recognition of this global heterogeneity in normal subjects between metabolism and perfusion, independent of the methods of normalization, using either PET alone or PET and SPECT is actually helpful rather than traditional thinking of being discordant, which requires application of different thresholds for diagnosis of functional abnormalities using PET or SPECT. It may offer additional diagnostic aid to visual interpretation and quantitative analysis when both metabolism and perfusion images are available and are correlated in analysis. This would be particularly important in the patients with subtle cortical abnormalities with different findings in perfusion and metabolism PET or SPECT perfusion and PET metabolic findings.

Although a large patient series is desirable, the regional heterogeneity of normal subjects derived from the current study nevertheless has demonstrated the differences between the kurtosis of metabolism and that of perfusion and thus resulting in difference of the regional heterogeneity as reflected in intra-subject correlations of perfusion versus metabolism. Moreover, the study did not suggest comparing inter-subject correlations of metabolism and perfusion across subjects for a particular region, which had been shown in the study to have marked variations, at least using the current automatic ROI generation.

As the study employs the intra-subject correlation between metabolism and perfusion for an individual subject, the results will not depend on a population mean or normal data set as conventionally used in statistical parametric imaging. The markers used to provide results of this study may provide potential diagnostic information when the combination of functional imaging modalities is available, as many brain diseases have deranged perfusion and metabolic coupling [8].

Finally, based on results here, a special use of markers based on global histogram features can be considered. In spite of the tight coupling between metabolism and perfusion that is physiologically based [3,8], histogram properties shown in the current study do differ quite significantly between these two PET imaging modalities. Though data here is limited to normals, it may be reasonable to expect that changes in histogram properties can possibly provide diagnostic capabilities among the varieties of brain diseases encountered. Further studies are ongoing to address this issue.

## Conclusion

Co-registered metabolism and perfusion revealed the regional heterogeneity that was encountered during intra-subject statistical comparison of the PET images. Correlation markers with attractively low coefficients of variation were found when using these two sets of images. Moreover the study here also revealed additional information content of the voxel histograms.

## Competing interests

The author(s) declare that they have no competing interests.

## Authors' contributions

CYOW carried out the design of the study, image coregistration and computer programming, participated in the statistical analysis and drafted the manuscript. JT carried out the statistical analysis and documentation, and drafted the manuscript. MG carried out the PET tracer administration and image acquisition. RP participated in the image reconstruction and transfer. JH and HYT participated in the database design, data downloading and web applications. DFW participated in the design of the study and helped the image analysis. HB, DFB and CN conceived of the study, and participated in its design and coordination and helped to draft the manuscript. All authors read and approved the final manuscript.

## Pre-publication history

The pre-publication history for this paper can be accessed here:


